# Hering: Domestic Physician

**Published:** 1883-05

**Authors:** 


### BOOK NOTICES.

HERING'S DOMESTIC PHYSICIAN, edited by C. R. Norton, M. D. Pp. 450. Price, $2.00. Philadelphia: Hahnemann Publishing House, F. E. Bœricke. 1883.

Dr. Hering's works on domestic practice form no small part of the history of Homœopathy. Very many are the converts to Homœopathy these works have made.

As a general rule, we cannot approve of the indiscriminate use of these books by parents. For it happens but too often that light cases of sickness are seriously aggravated by domestic prescribing; or perhaps the injury consists in the loss of invaluable time by such treatment. But in the country or in a locality where no good homœopathist lives this book is invaluable. As a case in point, we remember hearing of a lady who prescribed for her mother, in Paris, in a case so serious that two allopathists gave a gloomy prognosis. The lady carefully studied the case, gave the remedy, and in the morning the two regulars were very much surprised to see the improvement.

Not a few physicians could learn much from this work of Dr. Hering. There are many original and practical hints in it that will amply repay a perusal.

From the introduction we quote this good advice:

1. Always give but one remedy, and *only when this does no more good* another one.

2. When the patient, after having taken the medicine once, or oftener, *begins to feel better, however little, he should discontinue it,* lest the healthful progress of the cure be interfered with by taking too much; but as soon as the improvement ceases the same medicine should be taken again; or, in case the symptoms have altered, another more appropriate one.

The American Institute should buy copies of this domestic work for some of its leading men; they could learn much from it.

